# Indirect DNA Transfer and Forensic Implications: A Literature Review

**DOI:** 10.3390/genes14122153

**Published:** 2023-11-28

**Authors:** Francesco Sessa, Cristoforo Pomara, Massimiliano Esposito, Patrizia Grassi, Giuseppe Cocimano, Monica Salerno

**Affiliations:** 1 Department of Medical, Surgical and Advanced Technologies “G.F. Ingrassia”, University of Catania, 95121 Catania, Italy; cristoforo.pomara@unict.it (C.P.); monica.salerno@unict.it (M.S.); 2 Faculty of Medicine and Surgery, “Kore” University of Enna, 94100 Enna, Italy; massimiliano.esposito@unikore.it; 3 “Vittorio Emanuele” Hospital, University of Catania, 95121 Catania, Italy; patricgrassi@gmail.com; 4 Department of Mental and Physical Health and Preventive Medicine, University of Campania “Vanvitelli”, 80121 Napoli, Italy; peppecocimano1@gmail.com

**Keywords:** touch DNA, secondary transfer, forensic implications, indirect DNA transfer

## Abstract

Progress in DNA profiling techniques has made it possible to detect even the minimum amount of DNA at a crime scene (i.e., a complete DNA profile can be produced using as little as 100 pg of DNA, equivalent to only 15–20 human cells), leading to new defense strategies. While the evidence of a DNA trace is seldom challenged in court by a defendant’s legal team, concerns are often raised about how the DNA was transferred to the location of the crime. This review aims to provide an up-to-date overview of the experimental work carried out focusing on indirect DNA transfer, analyzing each selected paper, the experimental method, the sampling technique, the extraction protocol, and the main results. Scopus and Web of Science databases were used as the search engines, including 49 papers. Based on the results of this review, one of the factors that influence secondary transfer is the amount of DNA shed by different individuals. Another factor is the type and duration of contact between individuals or objects (generally, more intimate or prolonged contact results in more DNA transfer). A third factor is the nature and quality of the DNA source. However, there are exceptions and variations depending on individual characteristics and environmental conditions. Considering that secondary transfer depends on multiple factors that interact with each other in unpredictable ways, it should be considered a complex and dynamic phenomenon that can affect forensic investigation in various ways, for example, placing a subject at a crime scene who has never been there. Correct methods and protocols are required to detect and prevent secondary transfer from compromising forensic evidence, as well as the correct interpretation through Bayesian networks. In this context, the definition of well-designed experimental studies combined with the use of new forensic techniques could improve our knowledge in this challenging field, reinforcing the value of DNA evidence in criminal trials.

## 1. Introduction

In forensic investigations, sampling methods play a crucial role in obtaining DNA evidence. The careful collection of samples from crime scenes, victims, and suspects ensures the accuracy and reliability of DNA analysis [1]. At the same time, the extraction, quantification, and amplification of DNA from these samples further enhance the investigative process [2]. All these processes are vital as they enable forensic scientists to analyze and compare DNA profiles, aiding in the identification of individuals, linking suspects to crimes, and providing valuable evidence in court proceedings. In recent years, DNA profiling techniques have been developed into highly sensitive tools: to date, it is possible to obtain a complete profile using small quantities of DNA recovered at crime scenes (i.e., a complete DNA profile can be produced using as little as 100 pg of DNA, equivalent to only 15–20 human cells) [3,4,5,6]. In this context, on the one hand, several cold cases have been solved; however, on the other hand, it is possible to obtain a profile of a subject who was never physically at the scene. For these reasons, while defense attorneys rarely challenge the presence of DNA trace evidence (sub-source level) in court, they increasingly question the mechanisms of DNA transfer to the crime scene (activity level) [4,5,6].

The activity level of DNA transfer in criminal cases is of great importance as it has been observed that not only direct transfer of DNA (primary) can be found at a crime scene but also indirect transfer (secondary) from unrelated individuals through potential vectors such as objects or persons. Numerous studies have described this possibility, highlighting the crucial role that DNA transfer can play in criminal investigations [7,8,9,10]. A seminal paper on the possibility of indirect DNA transfer was written by van Oorschot and Jones in 1997 [11]. Fifteen years later, in another research paper on this theme, Daly et al. [12] reinforced the theory of Ladd et al. [13], describing the secondary transfer of DNA in two possible ways: from skin to skin to object or from the skin to object to skin.

Based on Locard’s exchange principle, which could be summarized with the sentence “every contact leaves a trace” [14], during a crime scene investigation (CSI), trace DNA may be collected from a suspected handled surface/object; based on a recent review, the so-called “touch DNA” could be composed of cell-free DNA, fragment-associated residual DNA, transferred exogenous nucleated cells, endogenous nucleated cells, or anucleate corneocytes [15,16,17,18]. The ability to release “touch DNA” may be subject-related. The first part of the research evaluated the ability to shed trace DNA, and forensic researchers concluded that a subject could be classified as a ‘good shedder’ or ‘poor/bad shedder’ [19,20]. Further studies clarified that on ‘shedder status’, not two but three categories of status should be used: high, intermediate, and low shedder [21,22].

In this scenario, numerous scientific works have investigated the phenomenon of ‘touch DNA’; however, the possibility of generating a ‘secondary transfer’ still remains a challenging scientific question that needs further investigation. For this reason, this review aims to provide an up-to-date overview of the experimental work carried out focusing on secondary DNA transfer, analyzing, for each selected paper, the experimental method, the sampling technique, the extraction protocol, and the main results. A critical overview of secondary transfer may be useful in order to define future research lines, filling the gaps in our knowledge in this challenging field.

## 2. Materials and Methods

### 2.1. Database Search Terms and Timeline

A systematic review was conducted according to the PRISMA guidelines [23].

Scopus and Web of Science (WOS) databases were used as the search engines from 1 January 1997 to 20 November 2023. The following keywords were used: (Touch DNA) AND (Secondary DNA Transfer); (Touch DNA) AND (Indirect DNA Transfer); (Touch DNA) AND (Secondary); and (Touch DNA) AND (Indirect). These keywords were searched within “Article title, Abstract, Keywords” for the Scopus database and “Topic” (searching within “Searches title, abstract, author keywords, and Keywords Plus”) for the WOS database.

### 2.2. Inclusion and Exclusion Criteria

For this literature review, only original articles, published in English, were included. On the contrary, articles not in English, reviews, letters, book chapters, conference papers, and notes were excluded in order to include only articles with a full description of the section about materials and methods. Similarly, any full research papers that were captured in the search but did not have this level of detailed method information were also excluded. Moreover, only articles that were in line with the study’s aim of reviewing indirect DNA transfer were analyzed. 

### 2.3. Quality Assessment and Data Extraction

All sources were screened for inclusion at both the title/abstract and full-text stages. All articles were first assessed by F.S.; then, M.S. conducted an independent re-analysis of the selected articles. If there were differing opinions concerning the articles, they were referred to C.P., who evaluated the criteria after reading the articles. Kappa’s statistical test [16] was used to gauge the level of agreement between the studies (Cohen’s Kappa = 0.92, demonstrating the strength of agreement between the included articles).

### 2.4. Characteristics of Eligible Studies

**Figure 1:**
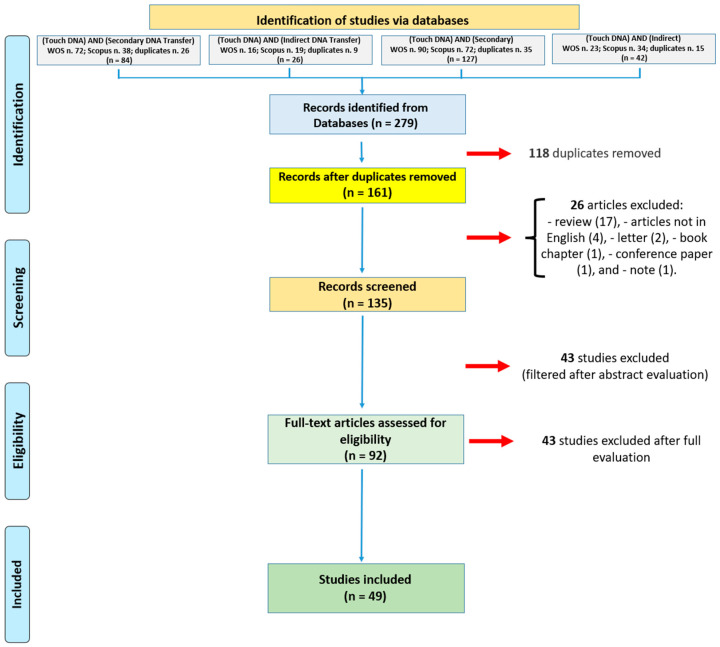
Flow diagram illustrating included and excluded studies in this systematic review.

As summarized in Figure 1, a total of 279 articles were obtained from the used databases. Of these, 118 duplicates were removed (using the automatic tool included in the Scopus database), and 26 studies were removed based on the exclusion criteria. Forty-three papers were then removed after abstract screening. After conducting a thorough evaluation, from the pool of 92 articles, 43 studies were excluded as they were not in line with the study’s aim. Ultimately, 49 articles were deemed suitable for the current systematic review.

## 3. Results

**Figure 2:**
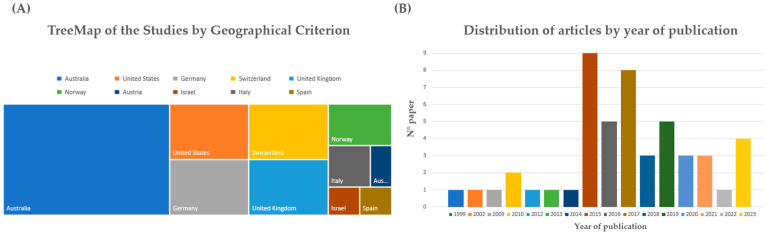
(**A**) TreeMap of the studies classified by geographical criterion. The distribution is based on the nationality affiliation of the first author of the study. (**B**) Distribution of articles by year of publication. The majority of the studies were published in the last seven years.

As summarized in Figure 2A, based on the first author’s affiliation, the research groups that contributed to the selected articles came from Australia (21), the United States (5), Germany (5), Switzerland (5), the United Kingdom (5), Norway (3), Italy (2), Austria (1), Israel (1), and Spain (1). Analyzing the distribution of articles by year of publication (Figure 2B), the first paper on indirect DNA transfer that included sufficient method details was published in 1999, while many studies were performed in the last seven years: 2002 (1), 2009 (1), 2010 (2), 2012 (1), 2013 (1), 2014 (1), 2015 (9), 2016 (5), 2017 (8), 2018 (3), 2019 (5), 2020 (3), 2021 (3), 2022 (1), and 2023 (4). 

The experimental model and the main results of the selected articles are summarized in Table 1.

### 3.1. Technical Results

Analyzing the main technical data (sampling method, DNA extraction, quantification, amplification), it is important to remark that all selected articles were performed over a wide period from 1999 to 2023, more than 20 years. In this period, forensic genetics constantly improved their methods, offering more sensitive and specific technologies that revolutionized this forensic field [61,62,63]. In general, the summarized results refer to DNA extraction, quantification, and amplification and are strictly related to the period when the study was performed (a study performed in 1999 did not have the possibility to use the same technologies as a study performed in 2023). The sampling method, the DNA extraction protocol, and the quantification and amplification techniques are summarized in Table 2. 

**Figure 3:**
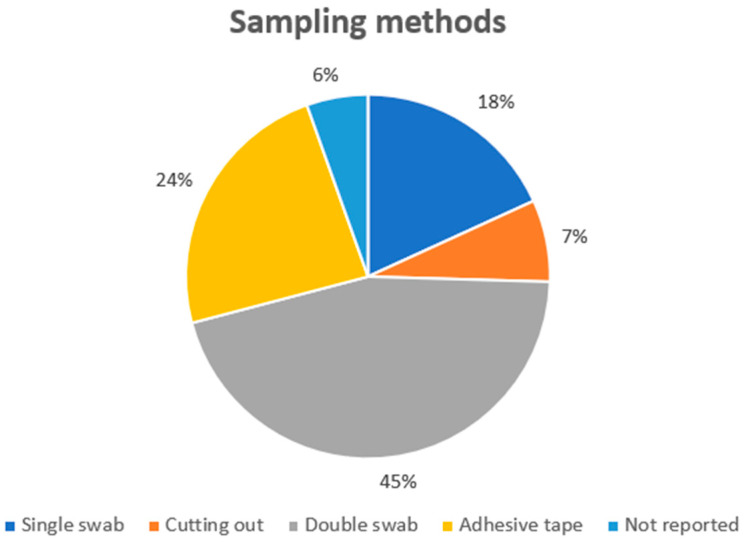
The sampling techniques applied in the selected studies.

Analyzing the sampling techniques (Figure 3), the most used technique to collect biological samples was the double swab (it was used in 25 cases), while the single swab was used in 10 experimental models. Adhesive tape was used in 13 cases, while cutting-out was used in four cases. Finally, three papers did not report the sampling method. The use of the double swab technique was justified by the experimental model: the main goal of each study was to focus on the secondary transfer generated after a touch. As reported in the literature and confirmed in this review, to sample skin cells, the single/double swab techniques, or adhesive tape, are the best methods to guarantee adequate cell recovery [16,71,72]. Moreover, the cutting-out technique could be applied in selected experimental models (i.e., garment sampling), considering that it may not always be used on hard surfaces. Regarding the sampling methods, a recent literature review concluded that the single-swab method showed the highest efficiency in touch DNA recovery in a wide variety of experimental settings [16]. 

**Figure 4:**
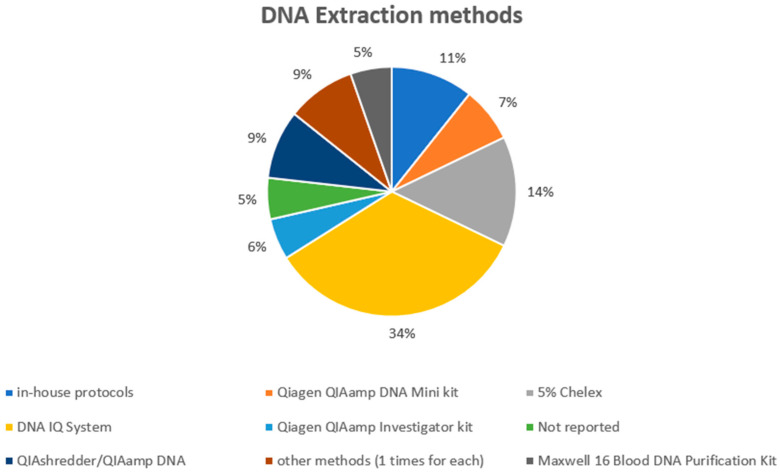
The DNA extraction methods applied in the selected studies.

Regarding DNA extraction (Figure 4), the DNA IQ System was the most used method (applied in 19 experimental models), followed by 5% Chelex (used in eight studies), while in-house protocols were applied in seven experimental models. The QIAshredder/QIAamp DNA extraction procedure was applied in five experimental models. Qiagen QIAamp DNA Mini kit (4), Qiagen QIAamp Investigator kit (3), Maxwell 16 Blood DNA Purification Kit (3), Qiagen DNA all-tissue DNA kit (1), “First-DNA” kit (2), PrepFiler Automated Forensic DNA Extraction Kit (1), and Speedtools DNA (1) were the techniques used in the other experimental articles. In three cases, the authors did not indicate the extraction protocol, while in six cases an “in-house method” was used. In a recent research article [71], it has been demonstrated that swabs and direct PCR could positively influence the DNA profiling from a touched item, reducing the number of required cells. 

**Figure 5:**
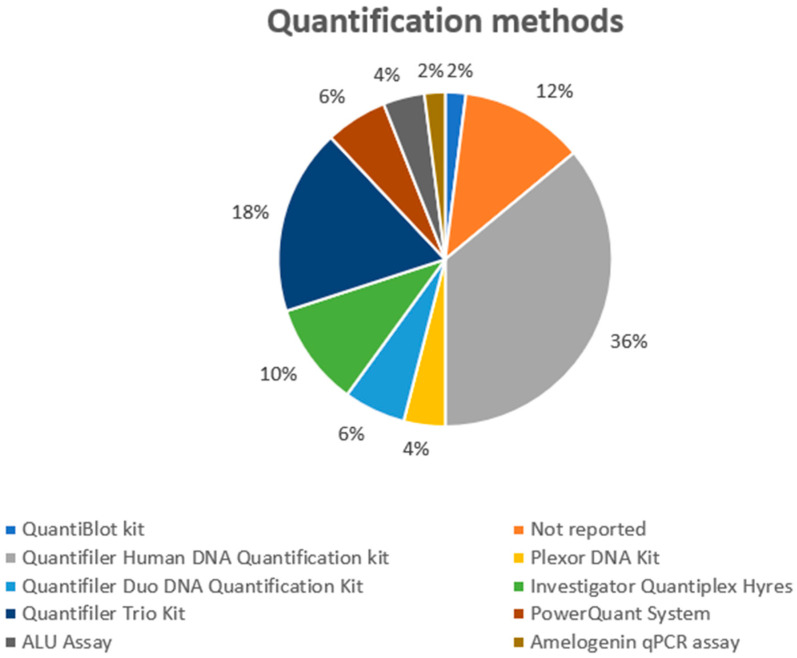
The DNA quantification methods applied in the selected studies.

Analyzing the quantification tool (Figure 5), in two articles, three quantification techniques were used in each, while a quantification technique was not used in two research articles; moreover, this information was not included in six experimental models. The kits used are listed below: Quantifiler Human DNA Quantification Kit was used in 18 studies, while Quantifiler Trio DNA Quantification Kit was applied in nine protocols. Quantifiler Duo DNA Quantification Kit (3), Investigator Quantiplex HYres (5), PowerQuant System (4), ALU assay (2), Plexor DNA Quantification Kit (2), QuantiBlot Kit (1), and Investigator Quantiplex Kit (5) were the other methods used. As previously described, modern techniques could improve profiling by applying direct PCR after swab sampling [71]. On the other hand, the use of quantification methods that may evaluate the quantification between male and female DNA, are very useful in the evaluation of activity level.

**Figure 6:**
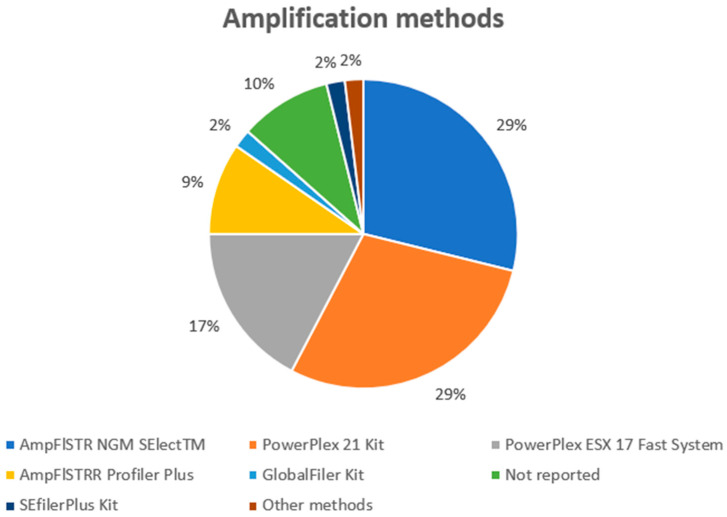
The amplification techniques used in the selected studies.

To perform genotyping (Figure 6), the most used kits were AmpFlSTR NGM SElectTM and PowerPlex 21 Kit (15), PowerPlex ESX 17 Fast System kit (10), AmpFlSTRR Profiler Plus (5), SEfilerPlus kit (1), and GlobalFiler (1). In one case, the authors reported that at least one of the following DNA amplification kits was used: AmpflSTR SGM Plus, SEFiler Plus, NGM Select (Life Technologies), PowerPlex ESX 17, ESI 17 (Promega), AmpflSTR Yfiler, or AmpflSTR Yfiler PLUS (Life Technologies) [48]. Finally, in four experimental models, the authors did not provide this information.

### 3.2. Main Findings

In the first paper published about secondary transfer, Ladd et al. [13] analyzed two possible ways to obtain a secondary transfer: skin to skin to object (handshaking) and skin to object to skin. Based on their results, the authors concluded that secondary transfer should be considered a very unusual event. Lowe et al. [24], in one of the first papers that investigated secondary transfer, reported that shedders may be distinguished into good and poor shedders and that secondary transfer (hand to hand to object) is more probable when the time interval is shorter. These authors concluded that secondary transfer under optimized conditions is possible and may result in a single full profile. As regards this concept, to date, shedder status no longer uses two categories of status but three: low, intermediate, and high shedder [21,22]. Szkuta et al. [54] reported the possibility of transferring DNA from the hand of a known contributor to another hand after a handshake, which could be subsequently transferred to, and detected on, a surface contacted by the depositor 40 min to 5 h post handshake. Jones et al. [41] demonstrated that it was possible to transfer DNA to a waistband and outside the front of underwear worn by a male following staged nonintimate social contact, while it is well described that intimate contact allows DNA transfer from the penis to underpants. Goray and van Oorschot [33] described that during daily activities, DNA may be transferred from one object to another, and in particular cases, the hand may be considered to be an indirect vector of the same DNA. Montpetit and O’Donnell [35] reported the possibility of finding foreign DNA on a cartridge after a gunshot, demonstrating the possibility of secondary transfer. Undoubtedly, the recovered touch DNA from fired cartridges is increasing thanks to the new technologies applied to forensic investigations both in sampling and profiling [73].

Szkuta et al. [38] demonstrated that secondary transfer is a possible event during laboratory procedures, demonstrating the potential for inter- and intra-exhibit contamination through further contacts. The same research group investigated different scenarios confirming the possibility of secondary transfer [37]. Goray et al. published two papers on the theme of secondary transfer, experimenting with different situations [25,26]. Their works were very important in clarifying several important aspects. Particularly, they clarified the importance of biological fluids in order to evaluate the possibility of the second transfer and the freshness of deposition; moreover, in the case of skin cells, it is important to evaluate the surfaces of the first and the second items. Moreover, they concluded that the secondary transfer is significantly influenced by the moisture content (i.e., in the case of wet substrates), item substrate type, and manner of contact (passive and pressure contact). In 2017, Szkuta et al. [50] reported that there was no correlation between the duration of handwashing and the extent to which self-DNA was transferred to the handprints of the depositors themselves or to those of the individuals who shook their hands. Taylor et al. [44] demonstrated in their experimental model that secondary transfer is a possible event in the workplace. They demonstrated that the DNA of individuals can be found in areas they do not frequent. This last event could be considered very hazardous because in similar cases it could be very difficult to establish if the subject is involved in a crime. Similarly, Onofri et al. [69] reported the possibility of a secondary transfer at a workplace from an object to another object, simulating a DNA transfer by means of the surface of a credit card. Considering that they found that the DNA transferred could be found as a major contributor, they justified their findings based on the surface (hard and non-porous surface), the time since deposition (fresh trace), and the type of contact (slight pressure and friction). According to Fonneløp et al. [31], it was demonstrated that DNA from the original user of computer equipment, such as a keyboard or mouse, can be transferred to the hands of a subsequent user up to eight days after receiving the items. Oldoni et al. [36] focused on the first and second handler of different items, reporting that after 120 min of handling or wearing objects, the majority of DNA found belonged to the second user. Despite this, the study focused on the first and second handlers, and the authors concluded that there is the possibility of an indirect transfer considering that they found external contributors. Cale et al. [40] described the effectiveness of secondary transfer on items, reporting that the texture of the item handled does not have a significant effect on DNA transfer. In line with these data, Fonneløp et al. [45] described the possibility of detecting foreign DNA on a t-shirt normally used without direct contact, demonstrating a secondary transfer from items. This probability was confirmed by Taylor et al. [52] and Samie et al. [43]. Obviously, the possibility to obtain a complete profile starting from a few cells thanks to new techniques has improved the possibility of detecting foreign DNA on an item that has never been touched.

McColl et al. [46] reported on the possibility of transferring saliva traces from one item to another item by hand, even if it is strictly related to different areas of the hand (i.e., palm, first finger).

Wiegand et al. [27] demonstrated the possibility of a secondary transfer from dried stains to gloves to other items, although it occurred under particular conditions. In this way, Neuhuber et al. [48] reported the possibility of a secondary transfer mediated by police officers during the detection or the analysis of items located at the crime scene. Indeed, as demonstrated by Thornbury et al. [65], indirect DNA transfer without physical contact with dried biological materials from various substrates is a possible event. Nevertheless, Tanzhaus et al. [64] demonstrated that although secondary transfer may be a possible reason for DNA to be found at a crime scene, it is a highly improbable event. A similar study was performed by Fonneløp et al. [32]: these authors showed that there are good and bad transfer items, as well as humans. Regarding the transfer condition, Warshauer et al. [28] reported that secondary transfer is more probable when biological fluid is not completely dried. In another study, Lehmann et al. [29] concluded that transfer is strictly related to the different items’ composition (for example, glass transferred better compared to other surfaces). In another study, Zoppis et al. [30] determined that transfer is more probable in relation to the body zone previously touched (i.e., sebaceous vs. non-sebaceous skin areas). Romero-García et al., 2019 [59], reported that hand washing can possibly reduce the amount of DNA deposited on items. Champion et al., 2019 [57], described the possibility of visualizing the cellular transfer through new applications such as fluorescent Diamond Dye (DD). The use of DD could be important because it does not influence DNA recovery. Otten et al., 2019 [58], reported the possibility of having a secondary transfer at a crime scene via working gloves, considering the shedder status of the suspect. Butcher et al. [56] described that for the analyzed item (knife), the regular user deposited significantly higher quantities of DNA than the second user and unknown sources, irrespective of contact duration. These results are in contrast with a similar study conducted by Pfeifer and Wiegand, 2017 [49], which concluded that the outcome depends mainly on the nature of the contact, the handle material, and user-specific characteristics. In accordance with this study, Gosch et al. [61] investigated four firearm handling scenarios, simulating different actions of the shooter. The amount of DNA after indirect transfer was strictly related to handling conditions and surface types of areas of the firearm. It is important to highlight the nature of the surface and the sampling techniques applied.

The study conducted by Oldoni et al. [42] found that an increase in second contact duration led to an overall negative correlation in the relative contribution of DNA between first and second users. Various unmonitored factors such as hand-washing frequency, previous object-handling activities, and the variable manner of contact can influence secondary transfer. Obviously, as remarked by Meakin et al. [47], when indirect transfer occurs, it decreases with increasing time between DNA deposition and recovery. 

Recently, Verdon et al. [39] investigated sampling techniques, concluding that there is no clear sampling method preference when attempting to differentially sample deposits of touch DNA layered over a pre-existing DNA background.

To investigate different scenarios, Voskoboinik et al. [55] tested the potential of laundry to generate DNA transfer, ascertaining the possibility of a secondary transfer through shared washing and mixing of new and used garments. These new data are in contrast with the results obtained by Kamphausen et al. [34]: in their experimental model, these authors demonstrated a possible secondary transfer between dirty clothes with biological fluids (i.e., blood cells) to another item, while they concluded that the secondary transfer generated from skin cells during a washing process is improbable. Ruan et al. [53] confirmed the opportunity for DNA transfer during regular laundry activities, demonstrating the opportunity for the acquisition of endogenous and foreign DNA during this process. Szkuta et al. [51] investigated the possibility of transferring trace DNA by reusing fingerprint brushes.

According to the experiments conducted by Szkuta et al., DNA transfer can occur during daily activities. The studies found that DNA from the person wearing a garment can accumulate in external areas, and individuals sharing the same space with the wearer can also contribute their DNA to the garment. In some cases, the wearer’s contribution may be minor or absent compared to their close associates, depending on the specific situation and the area of the garment [60,63]. Despite these important data, according to Samie et al. [62], the amount of DNA present on an item is primarily influenced by the handler’s deposition. They also found that in cases of secondary transfer, where the subject only touches the handler’s hand and not the object directly, the subject’s DNA was a minor contributor to the mixed profiles. Recently, Thornbury et al. [66] confirmed the possibility of a secondary transfer without physical contact from used clothing, e.g., through shaking. Similarly, Reither et al. [67] investigated two possible scenarios, demonstrating that an indirect DNA transfer could occur from clothing to flooring and from flooring to clothing in both ‘active’ and ‘passive’ situations, even if the DNA transfer was greater in active simulation. Interestingly, Monkman et al. [70] demonstrated that a domestic animal (in their experimental model they used a dog) could be a vector for human DNA transfer, demonstrating a transfer from the animal to a gloved hand during patting and a bed sheet while walking.

Carrara et al. [7] recently performed an experiment to investigate the possibility of generating an indirect transfer in burglary simulations, confirming this alarming event. McCrane and Mulligan [68] confirmed the possibility of an indirect transfer in their experimental model. In this study, a male and a female alternately held a pistol, and subsequently, the female’s hand was swabbed, demonstrating a secondary transfer. The study applied only a quantitative method to confirm the indirect transfer.

## 4. Discussion

Secondary DNA transfer is the process of transferring DNA from one object or person to another through an intermediary. For example, if two people shake hands and then one of them touches a knife, the DNA of the first person may be transferred to the knife through the second person. This phenomenon can have implications for forensic investigations as it can link innocent individuals to crime scenes or introduce foreign DNA to forensic samples. As previously described (Figure 2A), most of the articles (21 out of 49) were written by researchers from Australia, followed by the United States (5), Germany (5), Switzerland (5), and the United Kingdom (5). The other countries that had at least one article were Norway (3), Italy (2), Austria (1), Israel (1), and Spain (1). Despite the fact that this review focused only on research papers that have sufficiently detailed method sections, these results suggest that major efforts have been made by countries with common law legal systems; moreover, several countries such as Italy and Spain should improve their efforts in this challenging field. Analyzing Figure 2B, the research on secondary transfer DNA has increased in recent years, especially since 2015. The first article, with a description of the experimental model, was published in 1999, but only four more articles were published until 2010. From 2010 to 2023, there were 44 articles published, with the peak years being 2015 (9 articles), 2017 (8 articles), and 2019 and 2016 (5 articles). These data confirm that secondary transfer DNA is an emerging and relevant topic in forensic science, with a diverse and growing body of literature.

**Figure 7:**
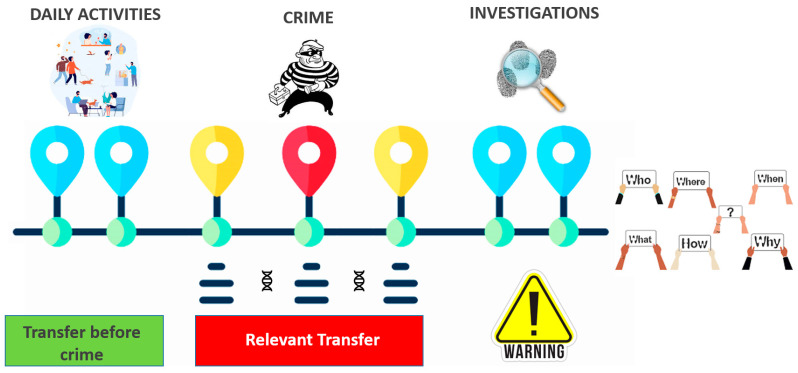
DNA can be transferred during various daily activities (green bar), but it is only significant when it comes to a crime involving items or persons. Different considerations must be made if the transfer takes place during a crime (red bar). Lastly, the transfer may be an unwelcome event during crime scene investigation or lab activities (warning), although it may be controlled following rigorous protocols.

As demonstrated in all experimental models, DNA transfer can occur anywhere during daily activities. This event becomes relevant in the case of a crime or when items are collected at a crime scene (Figure 7). Several cases of indirect transfer that had occurred in real criminal investigations were reported by Neuhuber et al. [48] who described indirect transfers via a camera, a car, and a desk, demonstrating the importance of being aware of this undesirable event.

In the last seven years, advancements have been made in genetic investigations in forensic sciences with the possibility of obtaining a complete DNA profile [32,74,75] and a forensic DNA phenotyping panel using massive parallel sequencing [76,77,78] with a small number of cells. In this context, the forensic laboratory has to establish the nature of the trace [79] as well as define reliable methods to establish the time since deposition [80]. To establish the nature of the trace and the time since deposition, transcriptome sequencing combined with biostatistical algorithms may be very useful in forensic cases [81,82,83,84]. Moreover, it is fundamental to clarify all aspects of indirect transfer as much as possible. Overall, the importance of sampling methods and the subsequent analysis of DNA cannot be neglected in forensic investigations as they serve as crucial tools in the pursuit of justice. As suggested by McCrane and Mulligan [68], using an inexpensive experimental model that does not require extensive technical expertise, it is possible to improve data in this research field, allowing for the participation of a wide range of laboratories and investigating a broad range of variables that could affect DNA transfer events.

Based on the results of the present review, in accordance with previous published reviews [85,86,87,88], the following variables should be considered in the evaluation of DNA transfer:

- The presence of DNA background: This refers to the amount and source of DNA that is already present on an object or surface before contact. A high DNA background can mask or dilute the secondary transfer, making it less likely to be detected [25,28,33,35,44,48,58,89].
- The subject’s characteristics: These include age, sex, shedder status (good or bad), and lifestyle habits. Some people tend to shed more DNA than others, which can affect the amount of DNA transferred and detected. Age and sex can also influence the quality and quantity of DNA, as well as lifestyle habits such as smoking, drinking, or using cosmetics [24,49,50,56,58,62].
- The type and duration of the contact: The type of contact can be direct (touching) or indirect (through an intermediary). The duration of contact can range from seconds to hours. Generally, direct and longer contacts are more likely to result in secondary DNA transfer than indirect and shorter contacts [24,29,32,36,40,43,55,57,63,69].
- The body zone previously touched: different body zones have different amounts and types of cells that can shed DNA, such as skin cells, sweat glands, hair follicles, or saliva glands. For example, touching the face or mouth can transfer more DNA than touching the arm or leg [30,34,38,40,59,61].
- The characteristics of the item: These include material, usage, size, shape, texture, and cleanliness. Different materials have different affinities for DNA, such as cotton being more absorbent than plastic. Usage can affect the amount of DNA background on an item, such as a frequently used phone having more DNA than a rarely used pen. Size, shape, and texture can affect the surface area and roughness of an item, which can influence the amount of contact and friction between the item and the DNA source. Cleanliness can affect the presence of contaminants or inhibitors that can degrade or interfere with DNA analysis [7,25,26,31,32,33,37,42,45,46,50,51,53,55,57,58,60,64,66,69].
- Trace type: This refers to whether the trace is fresh or dry, visible or invisible, single-source or mixed-source. Fresh traces are more likely to contain viable cells that can be amplified by PCR than dry traces. Visible traces are easier to locate and collect than invisible traces. Single-source traces are easier to interpret than mixed-source traces that contain DNA from multiple contributors [27,28,29,34,38,39,47,50,51,53,65,66,69].
- The activities made before contact: These include washing hands, wearing gloves, handling other items, or performing other actions that can affect the amount and quality of DNA on the hands or other body parts. Washing hands can reduce the amount of DNA available for transfer. Wearing gloves can prevent direct contact between the source and the target of DNA transfer. Handling other items can introduce additional sources of DNA or contaminants that can affect the analysis [30,33,34,38,40,59,61,63,68].

These factors are not exhaustive and may interact with each other in complex ways.

Other factors that could influence DNA transfer and its recovery are as follows:

- Time: The period of time between the primary and secondary contact and the interval between the secondary contact and the sampling of the evidence can affect the amount and quality of DNA transferred. Generally, the longer the time gap, the lower the chance of detecting secondary transfer DNA. However, there is no clear consensus on how long DNA can persist on different surfaces or objects after secondary transfer [36,38,40,41,42,46,54,56,63].
- Environmental conditions: The temperature, humidity, presence of microbial contamination, and other environmental factors can influence the degradation and persistence of DNA after secondary transfer. For example, high temperature and humidity can accelerate DNA degradation, while low temperature and humidity can preserve DNA for longer periods. Microbial contamination can also degrade DNA or interfere with its detection [32,33,37,41,44,47,52,53,60,65,68].
- Technical methods: The sampling methods, extraction methods, and profiling techniques used in forensic analysis can also affect the detection and interpretation of secondary transfer DNA. For example, different sampling methods (such as swabbing, taping, or cutting) can yield different amounts of DNA from the same surface or object. Different extraction methods (such as organic, Chelex, or silica-based) can be more efficient in isolating DNA from complex mixtures. Different profiling techniques (such as STRs, SNPs, or NGS) can have different sensitivities and specificities in amplifying and analyzing DNA from low-template DNA or degraded samples [38,39,42,48,52].

With this literature review, we aimed to clarify several important aspects of the techniques that could be used in order to improve results in this research field. On the contrary, we are unable to perform a data analysis of the analysis of the included papers because the experimental models are too varied and affected by different flaws. For example, several experiments did not perform the T0 swab on the hand/palm of the handler to verify the presence of exogenous DNA before starting the experimentation. As recently reported by Bini et al. [90], the use of alcohol-based hand sanitizer could reduce DNA transfer. 

Based on these findings, DNA transfer remains challenging in forensic science, both in case evaluations and in court testimony. Considering the results of this review that show the problems related to indirect transfer, it is more probable to obtain a DNA mixture from a piece of evidence. To assign the probability of DNA results, given competing propositions that specify the mechanisms of transfer, several factors must be considered to develop Bayesian networks to define DNA movement through complex transfer scenarios [91,92,93]. In this way, the analysis of biological traces found at crime scenes can rule out/include a possible suspect, providing a numerical estimate of the similarity between crime scene DNA and that of the suspect, obtaining a relatively high confidence score [94]. In this regard, in order to assess the value of forensic biological evidence, the DNA Commission of the International Society for Forensic Genetics (ISFG) published international guidelines highlighting the importance of activity-level propositions [95]. Nevertheless, as recently remarked by Kotsoglou and McCartney [96], the focus is on analyzing and assessing evidence shifts from the source to the activity, moving one step higher on the inferential ladder. This shift includes considering the mechanics of how the DNA sample was deposited, despite the fact that a significant portion of determining evidential sufficiency relies on establishing the source, which is the initial step in the hierarchy of propositions (source–activity–offense). This exercise is challenging, and the question remains whether a jury can draw a reasonable adverse inference. For these purposes, machine learning could be an optimal tool to evaluate the number of contributors in mixed profiles [97], as well as in the evaluation of complex Bayesian networks [91]. As regards these considerations, it should be taken into account that to date, the court is not always prepared to receive and interpret this kind of report to give the right “weight of evidence”. Recently, Morgan [98] reported that there is a call for forensic science to return to a scientific approach. The integration of legal requirements and research into forensic science practice and policy is seen as crucial. This author reported the importance of situating evidence within the entire forensic science process, developing an evidence base for each stage, and understanding the interaction of different lines of evidence. Earwaker et al. [99] remarked on this concept, confirming that it is necessary to minimize the misinterpretation of scientific evidence and maximize the effectiveness of crime reconstruction approaches and their application within the criminal justice system.

In this scenario, there are several open questions: how, when, and in which manner did the DNA arrive at a crime scene? First, laboratory personnel are called on to apply their skills to obtain DNA profiles starting from biological evidence, reducing/erasing potential contamination at every step. Individual hairs, sweat, and/or saliva inadvertently deposited by an investigator at a crime scene or during laboratory activities could cost valuable time, creating the risk of excluding a valid suspect, as well as misinterpreting physical evidence. In this context, indirect DNA transfer (also called secondary, tertiary, etc., transfer) of biological material via multiple steps (i.e., hand → hand → items, hand → item → hand, etc.) represents an event that could damage irremediably the investigation. Indeed, direct contamination could be limited by adopting the exclusion database containing reference profiles of subjects (police officers, healthcare personnel, etc.) involved in the CSI for automatic elimination, while its absence could favor contamination accidents [48]. This error, in addition to irreparably compromising the investigation, could lead to the conviction of a subject who was never at the scene, as pointed out by Tanzhaus et al. [64]. To eliminate both risks of contamination, the number of people present at the crime scene should be limited to well-trained personnel. Given that the potential for contamination of evidence (or the crime scene) increases as the number of people accessing the crime scene increases, there is an increasing need for the crime scene to be secured quickly by isolating and restricting access to it.

Another crucial aspect is the possibility that indirect transfer occurs during evidence packaging or laboratory activities [85,87]. New and sterile containers must be used to package all evidence, and the packaging equipment must also be free of contaminants. As largely discussed in this review, secondary transfer is a possible event both among different objects and among the same objects [31]. Indeed, indirect contamination could occur during evidence analysis, for instance at a forensic laboratory. This is another area for potential contamination: particularly, during sampling methods, an involuntary transfer may be carried out with sterile scissors or gloves [37,100]. Despite the presence of standard procedures for decontamination, analysts are aware of the risk of contamination and routinely clean their work areas. To minimize the potential risk of contamination, facilities and forensic scientists usually adopt standard procedures and policies. Therefore, it is crucial to perform decontamination procedures repeatedly during laboratory hours.

In this scenario, the value of DNA evidence in criminal trials should be re-evaluated. Scenarios involving multiple transfer events may increasingly account for the presence of a person’s genetic material at the crime scene. Considering what was previously discussed, the finding of genetic material is no longer sufficient to place that person at the crime scene. Without data on approximate transfer rates based on a set of variables, it is very difficult to estimate the probability of an outcome in each transfer event scenario. Given the paucity of well-designed studies on the matter, in accordance with Gosch and Courts [86], it is desirable that further research should be carried out after extensive literature research in order to understand the well-studied and under-researched transfer scenarios and the relative variables investigated (such as the sampling methods, the extraction protocol, and quantification and amplification kits). In particular, a set of new studies regarding secondary transfer could be focused on the poorly studied aspects, prioritizing the under-represented variables, questions, and scenarios. In this way, the use of ‘DNA-TrAC’ could be very useful as a guiding tool in the preliminary phase of each experimental study, despite the fact that it should be updated.

Lastly, several considerations should be made from an ethical point of view, considering that ethics should be an intrinsic part of a scientist’s daily practice in forensic genetics. Scientists should understand and act within ethical and legal boundaries, incorporating the operational and societal impacts of their daily decisions: particularly, considering indirect transfer as a possible event, every trace should be analyzed with attention [101]. Moreover, the retention of DNA samples and profiles by the police has been a subject of controversy, and this question could be amplified in the context of DNA transfer. The European Court of Human Rights (ECHR) has ruled that the ‘blanket and indiscriminate’ retention of DNA of individuals is disproportionate and breaches the European Convention on Human Rights. Under the new regime, DNA profiles of non-convicted individuals must be deleted after an investigation, with a maximum retention period of five years for those arrested or charged with qualifying offenses. Nevertheless, the impact of these limitations on the effectiveness of forensic DNA analysis remains unknown [102].

This review has several strengths, including a high value for Kappa’s statistical test, a wide temporal period analyzed, a detailed study selection process flowchart, and a comprehensive search methodology. However, there are also some limitations associated with the review. These include the possibility of selected keywords influencing the search strategy, potential influence from the author’s personal viewpoints, the inclusion of articles published only on WOS or Scopus, a small sample size that precludes complete statistical analysis, and gaps in literature searching practices that may be related to the use of selected databases. Moreover, this review included only research papers on indirect transfer that have sufficiently detailed method sections. Finally, it is important to remark that in order to perform a serious meta-analysis of data, the data should be obtained following well-defined procedures. On the contrary, the selected articles were extremely varied in their experimental model and methods, and the results were not always clearly or completely described.

## 5. Conclusions

In conclusion, secondary transfer is a complex and dynamic phenomenon that can affect forensic investigation in various ways. It depends on multiple factors that interact with each other in unpredictable ways. It requires careful methods and protocols to detect and prevent it from compromising forensic evidence. It has serious implications for forensic practice and justice that need to be addressed with awareness and education. The concern of law enforcement and forensic practitioners regarding the risk associated with evidence contamination dates back to the inception of evidence analysis. However, newer forensic analysis techniques have magnified the potential impact of contamination on criminal investigations due to the sensitivity of current forensic DNA analysis. Proper collection, packaging, handling during transport, storage, analysis, as well as decontamination procedures can significantly reduce the potential for contamination. At the same time, the possibility that a transfer occurs during daily activities represents a very hazardous event that could compromise DNA analysis.

In this scenario, the principal take-home message of this review is related to the different flaws of the published experimental models: therefore, it is necessary to highlight the importance of making well-designed studies, diminishing variability, in order to establish a solid scientific base for this insidious topic. The definition of well-designed experimental studies and the use of the most modern extraction and amplification techniques will make it possible to fill those gaps in our knowledge, reinforcing the value of DNA evidence in criminal trials.

## Figures and Tables

**Table 1:** The experimental method and the main results are summarized for each selected article.

Reference, Year, and Nationality	Experimental Model	Main Findings
Ladd et al., 1999, United States [13]	The researchers examined two forms of secondary transfer, which included skin to skin contact through handshaking and skin to object to skin contact.	Secondary transfer was not observed in this experimental model.
Lowe et al., 2002, United Kingdom [24]	In the first scenario, participants categorized as good and poor shedders were asked to hold hands for 1 min. Following this, poor shedders were instructed to hold a plastic 50 mL tube for 10 s.	Secondary transfer occurred when the DNA from the hand of the good shedder was transferred to an object through the poor shedder. The authors concluded that secondary transfer under optimized conditions is possible and may result in a single full profile.
	In the second scenario, there was a 30 min delay between the human contact and the poor shedders gripping the tube during the experiment.	In both the second and third scenarios, it was observed that the first shedder pairing led to secondary transfer. The recovered DNA profiles were mixed and included between 80 and 100% of the good shedder’s profile in every instance of secondary transfer.
	In the third scenario, there was a 1 h gap between the human contact and the tube gripping for the poor shedders in the experiment.	
Goray et al., 2009, Australia [25]	In this experimental model, three biological materials (pure DNA, blood, and saliva) were tested, evaluating the transfer between two different substrates: plastic (hard/non-porous) and cotton and wool (soft/porous). Wet samples were handled by depositing the biological fluid onto the primary substrate and then applying the secondary substrate within a time frame of 10–60 s. In the case of dry samples, the biological fluid was deposited onto the primary substrate and allowed to dry for 18–24 h (at room temperature) before the secondary substrate was applied. Contact was established through three modes: passive, pressure, and friction.	In this experimental model, it was found that the secondary transfer is significantly influenced by the moisture content (i.e., in the case of wet substrate), item substrate type, and manner of contact (passive and pressure contact). Although the experiment involved testing three distinct biological sources (pure DNA, saliva, and blood), there were no significant differences detected in the secondary transfer rates.
Goray et al., 2010, Australia [26]	The experiment involved creating a primary deposit of touch DNA by rubbing the skin over the designated area for 10–15 s. The researchers then evaluated the effects of different moisture levels (fresh and dry), primary and secondary substrates (soft porous cotton and hard non-porous plastic), and modes of contact (passive, pressure, and friction).	According to the experiment, the type of substrate used for the primary transfer plays a crucial role in the secondary transfer evaluation. If skin cells are deposited on cotton, the retrieved amount is approximately 20 times greater than when deposited on plastic. The secondary transfer of skin cells was found to occur more easily when the primary substrate was non-porous than porous. Contact through friction was also observed to significantly increase the rate of transfer.
Wiegand et al., 2011, Germany [27]	To conduct the experiment, 50 µL of saliva or venous blood from male donors were placed on paper, cotton cloth, and plastic surfaces, covering an area of 2 × 2 cm with equal moisture. The stains were left to air dry overnight and then subjected to two different scenarios for evaluation. The first scenario involved pressing the thumb on the stain for 2 s, while the second scenario involved rubbing the surface of a paper with the thumb for 10 s before swabbing the contact area to assess secondary transfer.	The transfer of saliva stains through rubbing and pressing onto paper only yielded 50% of detectable stains with very low levels of DNA (ranging from 0–1 pg/μL). On the other hand, the secondary transfer of blood stains resulted in relevant values. However, it was found that only a few instances of DNA concentrations were sufficient for complete DNA profiles, and these instances were transfers from stains on plastic.
Warshauer et al., 2012, United States [28]	First scenario: thumbs were licked, and subjects grasped sterilized plastic conical tubes after each drying time.	According to this study, when DNA is transferred from saliva, the genetic material of the original contributor can make up most of the resulting mixture. The amount of moisture present during the transfer, along with the texture and surface area of the object(s) involved, are important factors that affect the transfer. It is important to consider these factors when analyzing DNA transfers.
	Second scenario: saliva was deposited on pens; subsequently, subjects were required to pass the pens to their designated partners after each drying time; pens were gripped in the same way as the tubes; the partners’ palms were then swabbed.	
	Third scenario: thumbs covered by gloves were licked, and subjects grasped sterilized plastic conical tubes after each drying time; tubes were then swabbed.	
	In the fourth scenario of this study, pens were contaminated with saliva and then passed between subjects after different drying times. The pens were held like tubes and the subjects’ palms were moistened before they grasped them; the pens were swabbed for DNA analysis.	
	In the fifth scenario of this study, the subjects moistened their thumbs after each drying time by licking them. Following that, they grasped sterilized plastic conical tubes, which were then swabbed to collect any DNA.	
Lehmann et al., 2013, Australia [29]	This study involved depositing blood onto the substrate in 15 μL amounts and transferring touch DNA through various methods such as rubbing hands over cotton or repeatedly placing hands onto glass. Wet blood was immediately transferred while dry blood was allowed to dry completely. Touch DNA was deposited on the primary substrate and then transferred onto subsequent substrates within an hour. The primary substrate with DNA was flipped over and placed on top of the second substrate for deposition.	According to this study, the transfer of DNA from saliva is influenced by the substrate and the biological source types. The researchers confirmed the evidence of secondary and subsequent DNA transfer in their experimental model. They found that DNA transferred more readily to and from glass than it did to and from cotton. Additionally, the transfer of touch DNA was found to be less significant than wet or dry blood on either cotton or glass. Notably, the study found that wet blood transferred more effectively than dry blood on both cotton and glass surfaces.
Zoppis et al., 2014, Italy [30]	Three different scenarios were evaluated. First scenario: before handwashing, 8 subjects were asked to rub a fingertip on a typical sebaceous skin area of another individual (i.e., back of the hand and back of the forearm). Subsequently, they pressed on a glass slide. Second scenario: the same deposition was provided 10 min after conventional handwashing. Third scenario: the same deposition was provided 10 min after handwashing with antiseptic soap and air drying.	In evaluating genetic results, it is important to consider the specific previously touched cutaneous area, whether it is sebaceous or non-sebaceous skin areas, as DNA secondary transfer is a significant phenomenon.
Fonneløp et al., 2015, Norway [31]	In this study, each participant’s computer keyboard and mouse were exchanged with those of another participant, and the new user used the equipment for the entire duration of the study.	According to the findings of this study, it is feasible for the DNA of the first user to be transferred into the hands of a new user even eight days after the latter has touched the computer equipment.
Fonneløp et al., 2015, Norway [32]	The first substrate, either a piece of wood or a plastic tube, was picked up by the donors and held for 30 s with moderate pressure and friction. After this, the substrate was placed on a clean bench paper. The “investigator”, who wore personal protective equipment, then picked up the same substrate and held it in their right-hand glove for 30 s. Finally, the substrate was placed back onto the bench paper and the right-hand glove was held against new pre-cleaned items with moderate pressure and friction.	Based on the results of this study, DNA was readily transferred to wood and plastic, while less was transferred to a metal door handle, demonstrating that a second and tertiary transfer is a possible event.
Goray and van Oorschot, 2015, Australia [33]	During a 20 min social interaction, three individuals were invited to participate in a blind experiment where they had a drink of juice and chatted while being video recorded. The experiment involved collecting samples from various segments such as the table, chair arms (top only), jug handle, remaining surface of the jug, entire outer surface of the glasses, and left and right hands of each participant.	DNA that can be measured was discovered on numerous surfaces and objects during the testing. The lowest number of contributors necessary to account for the findings was recorded for each tested surface. In addition, some of the tested surfaces and objects exhibited unidentified DNA profiles.
Kamphausen et al., 2015, Germany [34]	The authors washed two pieces of clothing, one with skin cells and the other one with blood, either using a washing machine or hand-washing techniques.	According to the findings of the research, blood cells were consistently observed to transfer from one object to another. Combining buccal swabs and clothes for washing did not yield complete STR profiles. Lastly, the transmission of enough epithelial skin cells from one fabric to another during washing for a reliable STR analysis (i.e., a full profile) is highly unlikely.
Montpetit and O’Donnell, 2015, United States [35]	The goal of this study was to investigate the collection and profiling of DNA from both fired and unfired ammunition, which are frequently discovered during searches of individuals. To simulate various contact scenarios, DNA testing was conducted on casings and cartridges.	According to the findings of the current research, a combined profile was typically detected. However, in approximately 97% of cases, the individual operating the weapon’s ammunition loader was identified as the source of the profile.
Oldoni et al., 2015, Switzerland [36]	Various items, such as a computer mouse, pen, bracelet, necklace, key, watch, nurse cap, and nitrile gloves, were chosen for the study. The first participant used the objects frequently over a span of 8–10 days, while the second subject was asked to handle the same items for three separate simulation sessions of 5, 30, and 120 min each.	According to this study’s findings, the percentage contribution of the second user’s DNA profile increased significantly from 21% to 73% of the total DNA profile after 5 and 120 min, respectively, compared to the object’s owner on all objects examined.
Szkuta et al., 2015, Australia [37]	This study examined various situations to determine the extent and frequency of DNA transfer between simulated crime scene materials, such as cotton or glass, and high-risk vectors like scissors, forceps, and gloves.	According to this study, it was found that DNA-containing material could be transferred between exhibits through the use of scissors, forceps, and gloves. Touch DNA transfer was observed to be the highest when non-porous glass was used as the primary substrate, followed by porous cotton as the second substrate. These results demonstrate the potential for DNA transfer between different materials and objects and suggest that the source of the DNA profile may be identifiable even after transfer.
Szkuta et al., 2015, Australia [38]	In Experiment 1, dried blood or touch DNA was transferred from a primary substrate made of cotton or glass to a secondary substrate of DNA-free cotton or glass using scissors, forceps, or gloves. The researchers applied both heavy (multiple) and light (singular) contact in pairwise combinations. This implies that DNA can be transferred between exhibits through these common tools and that the source of the DNA profile may be identifiable even after transfer.	The authors concluded that a significant amount of DNA persisted on scissors, forceps, and gloves even after the transfer of dried blood from a primary cotton substrate to a DNA-free secondary cotton substrate. The nature of the contact did not impact the retention of dried blood on the vectors. However, the transfer of touch DNA from and to cotton resulted in fewer alleles remaining on these vectors.
Verdon et al., 2015, Australia [39]	Different scenarios were investigated (-touch/touch; -saliva/touch; -touch/saliva.	This study demonstrated that there is no clear preference for the sampling method, and the biological source is very important in order to determine the second transfer event.
Cale et al., 2016, United States [40]	The participants in this study wore gloves for 1.5 h before collecting samples to limit the presence of foreign DNA on their hands. Wearing gloves was also expected to promote the transfer of DNA by increasing the amount of sweat and oils on the participants’ hands. Once they removed the gloves, the participants shook hands vigorously for two minutes to simulate intimate contact, then immediately handled their assigned knife for two minutes.	The authors of this study were able to show that secondary DNA transfer, which refers to DNA belonging to an individual who did not directly touch the knife, was possible. Out of 20 instances, 16 were found to have alleles that could be attributed to this type of transfer.
Jones et al., 2016, United Kingdom [41]	In the first scenario, the male participant made physical contact with the female participant’s face for two minutes. Following that, both participants held hands and rubbed/massaged them together for three minutes. The male participant then proceeded to simulate urination by removing his penis from his underwear over the waistband and holding it with both hands for about 30 s. To increase the chances of DNA transfer, both hands were used to hold the penis before returning it to the underwear. Afterwards, the male volunteer removed his underwear while wearing gloves and swabbed the shaft of his penis.	On the underwear samples, DNA matching the female participant was detected. However, none of the penile samples taken 6 h after the staged non-intimate social contact events showed any matching female DNA.
	In the second scenario, the male participant collected penile swabs after engaging in unprotected sexual intercourse with a female. The researchers collected the underwear that the participant wore immediately after the intercourse and recovered the samples from it.	The female participant’s DNA profile was found to match on all waistband samples, inside front samples, and on samples collected from the inside back, outside front, and outside back areas of the clothing. These findings suggest a possibility of DNA transfer through physical contact with the female participant.
Oldoni et al., 2016, Switzerland [42]	The initial participant was instructed to interact with nine objects made of plastic, metal, nitrile, and fabric, which are typically present at crime scenes involving burglary or robbery. This interaction took place for at least 20 min per day, over a period of eight to ten consecutive days. Afterward, a second participant used the same objects in three different simulation sessions lasting 5, 30, or 120 min.	Indirectly transferred DNA accounted for only a small portion of the mixed DNA profiles observed, with the exception of 1 out of 234 cases.
Samie et al., 2016, Switzerland [43]	The objective of this study was to investigate the transfer of DNA from individuals with close connections to handlers.	Only a small percentage of the DNA profiles showed evidence of transfer from an unknown source, while the majority of profiles contained the DNA of the person who committed the stabbing.
Taylor et al., 2016, Australia [44]	These authors explore different work areas (laboratory areas, office areas, inaccessible areas, and common areas) in order to explore aspects of DNA transfer, including secondary and tertiary transfer.	Inaccessible areas were sampled to demonstrate secondary transfer. Moreover, these authors concluded that the detected profiles not always corresponded to the last person touching item.
Fonneløp et al., 2017, Norway [45]	Investigation on the secondary transfer using different scenarios in order to investigate possible occurrences of secondary transfer from co-workers (t-shirt used daily with investigation in order to detect an exogenous DNA profile)	These findings confirmed the possibility of obtaining a secondary transfer.
McColl et al., 2017, Australia [46]	The researchers utilized saliva from a male donor as a source of DNA that was manually transferred onto an object. To achieve this, four female participants pressed their dominant hand onto a plate coated with saliva for a period of 10 s and then immediately placed the same hand on a clean glass plate for another 10 s.	DNA transfer occurred in a different manner strictly related to the different parts of a hand.
Meakin et al., 2017, United Kingdom [47]	To mimic regular use, the researchers had each participant handle a knife in a specific way for two days in their experimental model. After that, the participants shook hands with a fellow volunteer for 10 s and then stabbed a foam block repeatedly with one of their knives for a minute.	With the exception of one participant, less than 5% of the recovered profiles had non-donor DNA co-deposited.
Neuhuber et al., 2017, Austria [48]	The authors investigated different scenarios (indirect transfer via camera; -indirect transfer via car; -indirect transfer via desk) about a police officer’s DNA transfer on crime scene samples, generating an indirect transfer as a source of contamination.	The authors confirmed the possibility of DNA transfer of police officers’ DNA onto crime scene items through three different scenarios.
Pfeifer and Wiegand, 2017, Germany [49]	In the first scenario, items belonging to one person are taken in a robbery by another person. In the second scenario, items are used by one person before being handled in a less severe manner.	When the second user simulated a burglary by using a tool barehanded, the first user may not be found as a major component on their handles. When the second user broke up the burglary setup using gloves, the first user matched the DNA handle profile in 37% of the cases.
Szkuta et al., 2017, Australia [50]	On glass plates, both the depositor’s self-DNA and non-self-DNA from the known contributor who shook hands with them were deposited.	The experimental model’s results indicate that a considerable amount of DNA is transferred, which is linked to an individual’s ability to transfer their own DNA (shedder status).
Szkuta et al., 2017, Australia [51]	The objective of this study was to assess the potential risk of contamination resulting from the transfer of dried saliva and skin deposits between glass surfaces using new, unused squirrel hair and fiberglass brushes. Various scenarios were examined during the investigation.	The experimental model’s results indicate that squirrel hair and fiberglass brushes can collect and transfer varying amounts of DNA-containing material. The detectability of the transferred material on the secondary surface depends on the biological nature of the material being transferred.
Taylor et al., 2017, Australia [52]	Transfer from hand to object, and, subsequently, secondary transfer from object to object.	Possibility to find the secondary transfer.
Ruan et al., 2018, Australia [53]	The researchers conducted a laundry experiment wherein 38 individuals were provided with a cotton swatch measuring roughly 10 cm × 10 cm to be washed and dried with their laundry using their own washing machine and detergent. As negative controls, two cotton swatches were randomly selected.	The DNA profiles of most cotton swatch samples indicated either a distinct single source (21%) or a blend of DNA from multiple sources (55%). In the case of mixed profiles, the majority of them (around two to three persons) showed DNA from only one source, while a few (around one in five) had a combination of DNA from four individuals.
Szkuta et al., 2018, Australia [54]	After exchanging two handshakes, the participants resumed their regular activities for either 40 min, 5 h, or 8 h. Later, they held a polished wooden axe handle with their right hand and rotated it to produce friction for 10 s.	The profiles obtained from the axe handles after they were in contact with the known contributor for 40 min, 5 h, or 8 h showed a diverse range of alleles. In all the profiles from the axe handle, except for one four-person mixture generated after the depositor’s contact 40 min post-handshake, the depositor was the primary or sole contributor.
Voskoboinik et al., 2018, Israel [55]	Under various washing conditions, a group of eight new socks made of different cotton blends were washed together with the regular laundry of four households.	The possibility of a secondary transfer was confirmed by 7/32 samples (22%).
Butcher et al., 2019, United Kingdom [56]	The researchers designed an experiment where a person used knives for 4 min over two days before another individual used them for 2, 30, or 60 s to determine how shorter durations of second use affect the resulting DNA profiles.	The DNA ratios of the first user to the second user were around 4:1, 2:1, and 1:1 for durations of 2, 30, and 60 s respectively. The analysis of the DNA quantities showed that the trend occurred due to a decrease in the DNA of the first user, transferred to the second user’s hands, rather than an increase in DNA deposition from the second user. This trend was observed after the knives were used by the first user for a total of 4 min over two days before being used by the second user for the specified durations.
Champion et al., 2019, Australia [57]	In this research, the contact types adopted by Goray et al. [18], namely, passive, pressure, friction, and friction with pressure, were employed to explore the transfer between aluminum and the substrates.	For the first time, researchers were able to visually detect the transfer of DNA from one substrate to another by using fluorescent Diamond™ Dye (DD) to visualize the cellular transfer.
Otten et al., 2019, Germany [58]	In this study, the goal was to evaluate the extent to which the DNA of an innocent person is transferred to a crime scene through work gloves, taking into account whether the suspect is a shedder or not.	The results of this study showed that the glove, especially its exterior, could act as a vector for secondary transfer in real-life scenarios.
Romero-García et al., 2019, Spain [59]	The researchers instructed five individuals to hold hands for five minutes and then wash their hands with soap having a neutral pH level. Next, they dried their hands using different towels each day. To serve as a control group, the researchers analyzed a portion of each towel that was not used, and they also collected saliva samples from all participants to determine their reference profiles.	The researchers were unable to obtain a comprehensive profile from either the towel or the individual who had made contact with the object, as well as their partner.
Szkuta et al., 2019, Australia [60]	This study examined various situations involving four participants, also known as “wearers” (P1-4). Two upper garments that belonged to the primary participant and had been worn before were chosen. Each of the selected garments was worn on both a workday (WD) and a non-workday (ND).	The research suggests that in certain situations, the presence of close associates may overshadow the wearer’s contributions, depending on the situation and the area of the garment. As a result, the wearer’s contributions may be minor or even absent.
Gosch et al., 2020, Germany [61]	Four firearm handling scenarios, simulating different actions of the shooter.	The amount of DNA subsequent to indirect transfer is strictly related to handling conditions and surface types.
Samie et al., 2020, Switzerland [62]	For all the experiments, a pair of identical knives were utilized, with one assigned to each participant. The participants, categorized as either a good or bad shedder, were instructed to shake hands before using their respective knives to stab the ballistic soap.	The secondary transfer is related to the shedder status, target surfaces, and alleged transfer mechanisms. Compared to the primary transfer, it occurs with a percentage between 1 and 3%.
Szkuta et al., 2020, Australia [63]	This study recruited four participants, also referred to as “wearers,” from four different laboratories. The participants wore the selected garments for an average of 5.1 h before hugging another individual, an average of 5.2 h (minimum 3.5 h, maximum 6.5 h) before going out with another individual, and an average of 1.5 h before spending a day in another individual’s office. The time spent wearing the garments includes both at home, during commuting, and at work before engaging in the respective activity.	According to this study, the DNA of a person of interest was successfully recovered from a piece of clothing after direct contact, close proximity, and physical absence. The experiment involved embracing an individual or occupying their office space, after which several DNA profiles were identified from the clothing. The transfer of DNA was more likely to occur following prolonged and/or recurring contact, as well as direct contact.
Tanzhaus et al., 2021, Germany [64]	This study involved the use of gloves of various materials such as cloth, leather, and rubber, which were sorted based on the material present on the exterior of the glove. The gloves were kept in separate plastic bags and handled by a perpetrator for a period of 4 weeks. Following each touch, the item was tested for DNA transfer.	Out of all the experiments conducted in this study, it was found that only one instance of secondary transfer could be detected.
Thornbury et al., 2021, Australia [65]	This study aimed to investigate the potential of DNA transfer without direct contact by analyzing tapping and stretching agitation for dried blood, saliva, semen, touch, and vaginal fluid that were deposited on four substrates.	This study found that it was possible for DNA to be transferred indirectly without any physical contact, as long as dried biological materials were present on different surfaces. The success of this transfer seemed to depend on various factors, such as the type of agitation, the type of biological material, and the surface it was transferred onto.
Thornbury et al., 2021, Australia [66]	This study focused on exploring the possibility of DNA transfer through indirect means without physical contact. The researchers achieved this by gently shaking used clothing, pillowcases, and towels, all of which had a known usage history, of 10 volunteers to check for DNA transfer onto a secondary surface. The results indicate that DNA transfer was a common occurrence and could take place from all three items that were tested.	This study’s experimental model revealed that DNA transfer to the secondary surface was observed in all samples except for four.
Reither et al., 2022, Australia [67]	The authors investigated two possible scenarios of indirect transfer: from a worn garment to a floor and vice-versa.	Based on their findings, the authors demonstrated the possibility of an indirect DNA transfer from clothing to flooring and from flooring to clothing in both ‘active’ and ‘passive’ situations. Obviously, the DNA transfer was greater in the active simulation (i.e., application of pressure and friction).
Carrara et al., 2023, Switzerland [7]	This study investigated a secondary transfer mediated by gloves during simulated burglary simulations.	This study confirmed the possibility of an indirect transfer in the applied experimental model.
McCrane and Mulligan, 2023, USA [68]	Different scenarios were investigated: a male and female alternately held the pistol, and subsequently, the female’s hand was swabbed to evaluate the secondary transfer.	Possibility to indirectly transfer the DNA on the female’s hand.
Onofri et al., 2023, Italy [69]	The authors performed a secondary transfer scenario simulating that the owner of a credit card, after his personal use for a month, places and moves it around the surface of a co-worker’s desk, applying slight pressure, for 30 s.	The authors reported a high value of secondary transfer (about 50% of secondary transfer DNA traces), and it was demonstrated that the co-worker could be identified as the major contributor.
Monkman et al., 2023, Australia [70]	The authors explored the possibility of an indirect transfer mediated by a domestic dog.	Based on their findings, the authors concluded that dogs could be a vector for human DNA transfer, demonstrating a transfer from the dog to a gloved hand during patting and a bed sheet while walking.

**Table 2:** The sampling technique, the extraction protocol, and the quantification and amplification techniques are summarized for each selected article.

Reference	Sampling Methods	DNA Extraction	Quantification	Amplification
Ladd et al. [13]	Moistened (dH_2_O) sterile swabs.	Organic extraction/microcon-100 purification, following in-house protocol.	QuantiBlot Kit (Perkin Elmer Applied Biosystems, Shelton, WA, USA).	AmpFlSTRR Profiler Plus and COfiler DNA typing kits (Perkin Elmer Applied Biosystems).
Lowe et al. [24]	Swabs of surfaces.	Qiagen QIAamp DNA Mini Kit (Qiagen, Hilden, Germany).	DNA was not quantified.	AmpFlSTRR Profiler Plus and COfiler DNA typing kits (Perkin Elmer Applied Biosystems).
Goray et al. [25]	The 1 cm × 1 cm small squares (plus a surrounding margin of approximately 0.3 cm) were cut into smaller pieces and placed into 10 mL tubes.	DNA was extracted via 5% Chelex.	Quantifiler Human DNA Quantification (Perkin Elmer Applied Biosystems).	AmpFlSTRR Profiler Plus kit (Perkin Elmer Applied Biosystems).
Goray et al. [26]	The 1 cm × 1 cm small squares (plus a surrounding margin of approximately 0.3 cm) were cut into smaller pieces and placed into 10 mL tubes.	DNA was extracted via 5% Chelex.	Quantifiler Human DNA Quantification (Perkin Elmer Applied Biosystems).	AmpFlSTRR Profiler Plus kit (Perkin Elmer Applied Biosystems).
Wiegand et al. [27]	Cotton wool swabs moistened with sterile water.	First-DNA all-tissue DNA kit (GEN-IAL GmbH, Troisdorf, Germany) DNA IQ extraction protocol (Promega, Madison, WI, USA).	Plexor DNA Quantification Kit (Promega).	SEfilerPlus kit (Applied Biosystems, Waltham, MA, USA).
Warshauer et al. [28]	Swab.	Qiagen QIAamp DNA Mini (Qiagen).	Quantifiler Human DNA Quantification Kit (Life Technologies, Carlsbad, CA, USA).	AmpFlSTR Identifiler Plus PCR Amplification Kit (Life Technologies).
Lehmann et al. [29]	This study involved cutting-out cotton substrates and plastic backing using scalpels and then extracting them together. To collect DNA from glass slides, the researchers used a double swab technique where the first swab was moistened with 4 drops of deionized water, and the second swab was slightly dampened with one drop of water.	DNA IQ Automated DNA extraction Kit (Promega).	Quantifiler Human DNA Quantification Kit (Life Technologies).	PowerPlex1 21 Kit (Promega).
Zoppis et al. [30]	The slides were swabbed with sterile cotton swabs and distilled water.	DNA IQ Automated DNA extraction Kit (Promega).	Quantifiler Duo DNA Quantification Kit (Applied Biosystems).	AmpFlSTR1 NGM SElectTM PCR Amplification kit (Applied Biosystems).
Fonneløp et al. [31]	Samples were collected by swabbing the participants’ hands.	All samples were extracted by 5% Chelex.	Quantifiler Duo Kit (Thermo Fisher, Waltham, MA, USA).	PowerPlex ESX 17 Fast System kit (Promega).
Fonneløp et al. [32]	DNA was recovered from all items using DNA-free mini-lifting tapes (Scenesafe FAST).	DNA was extracted by the 5% Chelex.	Quantifiler Duo Kit (Applied Biosystems).	Powerplex ESX 17 Kit (Promega).
Goray and van Oorschot [33]	The wet and dry double swabbing technique was used.	DNA IQ System (Promega).	Quantifiler Human DNA Quantification Kit (Thermo Fisher).	PowerPlex1 21 System (Promega).
Kamphausen et al. [34]	Dried clothes were taped with self-adhesive tape, and cells were collected from the tape with a double swab technique using first a DNA-free swab, moistened with lysis buffer, and then a dry swab.	DNA extraction from artificial stains was performed using a modified phenol/chloroform method.	Quantifiler Human DNA Quantification Kit (Thermo Fisher).	Powerplex ESX 17 or Powerplex S5 Kit (Promega).
Montpetit and O’Donnell [35]	The cartridges or casings were swabbed using a single nano pure water moistened cotton-tipped swab.	BioRobot EZ1 (Qiagen) using the EZ1 DNA Investigator Kit.	Quantifiler Human DNA Quantification Kit (Thermo Fisher).	AmpFlSTR Identifiler Plus PCR Amplification Kit (Life Technologies).
Oldoni et al. [36]	Samples were collected either with the double swab technique or by direct object cutting (nurse cap).	DNA was manually extracted using the QIAshredder/QIAamp (Qiagen) kit or phenol/chloroform (nurse cap).	Investigator Quantiplex HYres (Qiagen).	AmpFlSTR NGM SElectTM PCR Amplification (Life Technologies).
Szkuta et al. [37]	The wet–dry swabbing technique was used to collect samples from glass slides.	DNA IQ™ (Promega, USA).	Quantifiler Human DNA Quantification Kit (Thermo Fisher).	PowerPlex 21 System (Promega).
Szkuta et al. [38]	The wet and dry double swabbing technique was used.	DNA IQ System (Promega).	Quantifiler Human DNA Quantification Kit (Thermo Fisher).	PowerPlex1 21 System (Promega).
Verdon et al. [39]	Two different tapelift types were used: Scenesafe FAST, and Scotch Magic tape.	Following pre-treatment with 500 μL of TNE buffer containing Proteinase K, DNA was extracted from tapes and substrates using the DNA IQ system (Promega).	Quantifiler Human DNA Quantification Kit (Thermo Fisher).	PowerPlex 21 (Promega).
Cale et al. [40]	The surface of each knife’s handle was immediately sampled using a wet swabbing technique.	The process of removing the swabs from both the smooth-handled and rough-handled knives, as well as the control swabs, was carried out using the DNA Purification from Buccal Swabs Spin Protocol by Qiagen, a company based in Hilden, Germany. (Hilden, Germany).	Quantifiler Human DNA Quantification Kit (Thermo Fisher).	AmpFlSTR Identifiler Plus PCR Amplification Kit (Life Technologies).
Jones et al. [41]	The researchers used a wet sterile cotton swab that had been moistened with deionized water, followed by a dry sterile cotton swab to collect the DNA. They also took samples from specific areas of the underwear using mini-tape, including the inside and outside of the front waistband, as well as the inside front panel.	Not reported.	Not reported.	Not reported.
Oldoni et al. [42]	DNA traces were collected using the double swab method, except for the fabric nurse cap (cutting-out).	Within 24–48 h of sample collection, the DNA was extracted manually from the traces using the QIAshredder/QIAamp DNA mini protocol (Qiagen AG, Basel, Switzerland).	Investigator Quantiplex HYres (Qiagen).	AmpFlSTR NGM SElectTM PCR Amplification (Applied Biosystems).
Samie et al. [43]	DNA was collected using the double swab method.	DNA was extracted, using the combination of two kits, QIAshredder and QIAAmp kit.	Investigator Quantiplex HYres (Qiagen).	NGM Select (Applied Biosystem-Life Technologies).
Taylor et al. [44]	The sampling method used depended on the surface being sampled. Non-porous surfaces were sampled using foam-headed swabs called popule swabs that were soaked with isopropanol during sampling. Porous surfaces, on the other hand, were sampled using tapelifts.	DNA IQ system (Promega) using in-house validated protocols.	Quantifiler Human DNA Quantification Kit (Thermo Fisher).	GlobalFiler (Thermo Fisher).
Fonneløp et al. [45]	The mini-tape (Scenesafe FAST™) was used.	After sampling, the mini-tape was fragmented into smaller pieces and transferred to an extraction tube, from which DNA was extracted using the 5% Chelex method.	Quantifiler Trio Kit (Applied Biosystems).	PowerPlex ESX 17 Fast System kit (Promega).
McColl et al. [46]	DNA was collected using the double swab method.	DNA IQ™ System (Qiagen).	Quantifiler Human DNA Quantification Kit (Life Technologies).	PowerPlex 21 (Promega).
Meakin et al. [47]	DNA was recovered by mini taping.	QIAamp DNA Investigator Kit (QIAgen).	Quantifiler Human DNA Quantification Kit (Applied Biosystems).	AmpFlSTR NGM SElectTM PCR Amplification Kit (Applied Biosystems).
Neuhuber et al. [48]	Not reported.	“First-DNA” kit (Genial), M48 robot (Qiagen), or by organic extraction (phenol/chloroform).	Not reported.	Different DNA amplification kits.
Pfeifer and Wiegand [49]	The tool handles were cleaned using premoistened swabs (Sarstedt) soaked in lysis buffer (Promega).	The extraction of all samples was carried out using the Maxwell 16 Blood DNA Purification Kit (Promega) in a Maxwell extraction system.	Plexor HY System (Promega).	PowerPlex ESI 17 Fast (Promega).
Szkuta et al. [50]	Cotton swabs (150C, Copan) were utilized with a wet/moist swabbing protocol to collect the deposits on glass plates.	DNA IQ™ (Promega, USA).	Quantifiler Human DNA Quantification Kit (Life Technologies).	PowerPlex 21 (Promega).
Szkuta et al. [51]	DNA was obtained from swabs and cut bristles.	DNA IQ™ (Promega).	Quantifiler Trio (Life Technologies).	PowerPlex 21 (Promega).
Taylor et al. [52]	Double swab method and tapelifts (using Scotch Magic and Scenesafe FAST).	QIAshredder and QIAAmp kit.	Not reported.	Not reported.
Ruan et al. [53]	A DNA tapelift kit (Lovell Surgical Supplies) was used.	The DNA extraction process involved placing the tape inside an AutoLys tube manufactured by Hamilton Company, USA, followed by extraction using the PrepFiler Automated Forensic DNA Extraction Kit from Thermo Fisher Scientific.	Quantifiler Human DNA Quantification Kit (Applied Biosystems).	PowerPlex 21 (Promega).
Szkuta et al. [54]	Deposits on the axe handle were collected using a wet-moist swabbing technique.	Not reported.	Quantifiler Trio (Life Technologies).	Not reported.
Voskoboinik et al. [55]	Three-layer adhesive tapes were used to sample all garments.	Chelex extraction and subsequent purification with DNA IQ kit (Promega).	Quantifiler Human DNA Quantification Kit (Applied Biosystems).	AmpFlSTR SGM Plus (Applied Biosystems).
Butcher et al. [56]	DNA was recovered from each knife handle using a mini-tape within an hour of each stabbing event.	DNA extractions were performed using the QIAamp DNA Investigator Kit (Qiagen).	Quantifiler Human DNA Quantification Kit (Applied Biosystems).	AmpFlSTR NGM SElect™ PCR Amplification Kit (Applied Biosystems).
Champion et al. [57]	Not performed.	Not performed.	Not performed.	Not performed.
Otten et al. [58]	To collect DNA from items, sterile swabs were moistened with HPLC grade water.	DNA extraction was performed using a Maxwell16 Forensic Instrument with Casework Extraction Kit and DNA IQ™ Casework Pro Kit (Promega).	PowerQuant System (Promega).	PowerPlex ESX 17 System (Promega).
Romero-García et al. [59]	Not reported.	DNA was extracted with Speedtools DNA extraction kit (Biotools).	Not reported.	AmpFℓSTR NGM Select Kit (Thermo Fisher).
Szkuta et al. [60]	Polyvinyl chloride tape stubs (in-house). Mini-tape (SceneSafe FAST). Mini-tape (SceneSafe FAST). Moistened cotton swab followed by dry cotton swab (150C, Copan).	QIAmp isolation (Qiagen). Chelex (modified method). EZ1 advanced XL (Qiagen). DNA IQ™ (Promega).	ALU assay (in-house); Quantifiler HP (Thermo Fisher Scientific); Quantifiler Trio (Thermo Fisher Scientific).	NGM (Thermo Fisher Scientific); PowerPlex ESX16 Fast (Promega); -NGM SElect (Thermo Fisher Scientific); PowerPlex 21 (Promega).
Gosch et al. [61]	Modified ‘double swab’ technique.	In-house method.	PowerQuant System (Promega GmbH).	PowerPlex ESX 17 Fast Kit.
Samie et al. [62]	After the stabbing, both knife handles were swabbed for DNA using a single moist COPAN’s FLOQSwab, covering their entire surface.	Using both the QIAshredder and QIAamp DNA mini kit from Qiagen, DNA was collected from the swabs.	Investigator Quantiplex Kit (Qiagen).	NGM SElect (Applied Biosystem).
Szkuta et al. [63]	Polyvinyl chloride tape stubs (in-house). Mini-tape (SceneSafe FAST™). Mini-tape (SceneSafe FAST™). Moistened cotton swab followed by dry cotton swab (150C, Copan).	QIAmp isolation (Qiagen). Chelex (modified method). EZ1 advanced XL (Qiagen). DNA IQ™ (Promega).	ALU assay (in-house); Quantifiler HP (Thermo Fisher Scientific); Quantifiler Trio (Thermo Fisher Scientific).	NGM (Thermo Fisher Scientific); PowerPlex ESX16 Fast (Promega); -NGM SElect (Thermo Fisher Scientific); PowerPlex 21 (Promega).
Tanzhaus et al. [64]	Secondary transfer surfaces were swabbed with DNA-free cotton swabs.	Promega Maxwell RSC 16 robot (Promega) with the Promega Maxwell RSC custom total nucleic acid kit.	PowerQuant system (Promega).	Powerplex ESX 17 fast and Powerplex ESI 17 fast (Promega).
Thornbury et al. [65]	Wet and dry double swabbing using cotton swabs (Copan) and wetting with a few drops of sterile distilled water was applied to collect DNA from different surfaces.	DNA IQ (Promega, USA).	Quantifiler Trio (Applied Biosystems).	PowerPlex 21 System (Promega).
Thornbury et al. [66]	Wet and dry double swabbing using cotton swabs (Copan).	DNA IQ System (Promega).	Quantifiler Trio DNA Quantification Kit (Applied Biosystems).	PowerPlex 21 System (Promega).
Reither et al. [67]	Wet and dry double swabbing.	DNA IQ system (Promega).	Quantifiler Trio DNA Quantifica-tion Kit (Applied Biosystems).	PowerPlex 21 System (Promega)
Carrara et al. [7]	Wet and dry double swabbing.	QIAshredder/QIAamp DNA extraction procedure (Qiagen).	Investigator Quantiplex HYres Kit (Qiagen).	AmpFLSTR NGM SElect PCR amplification kit (Life Technologies).
McCrane and Mulligan [68]	Single swab.	Isohelix XME-50 Xtreme DNA Isolation kit (Midwest Scientific, Fenton, Missouri).	Samples were tested using the Amelogenin qPCR assay.	Not performed.
Onofri et al. [69]	Adhesive tape.	Phenol–chloroform organic method.	PowerQuant System (Promega).	PowerPlex ESX17 Fast kit (Promega).
Monkman et al. [70]	Wet–dry swabbing technique.	DNA IQ system (Promega).	Quantifiler Trio DNA Quantifica-tion Kit (Applied Biosystems).	PowerPlex 21 System (Promega)

## Data Availability

Data are contained within the article.
